# Effects of DNA mass on multiple displacement whole genome amplification and genotyping performance

**DOI:** 10.1186/1472-6750-5-24

**Published:** 2005-09-16

**Authors:** Andrew W Bergen, Ying Qi, Kashif A Haque, Robert A Welch, Stephen J Chanock

**Affiliations:** 1 Division of Cancer Epidemiology and Genetics, National Cancer Institute, National Institutes of Health, Bethesda, MD, USA; 2 Core Genotyping Facility, National Cancer Institute, National Institutes of Health, Gaithersburg, MD, USA; 3 Intramural Research Support Program, SAIC-Frederick, NCI-FCRDC, Frederick, MD, USA; 4 Section on Genomic Variation, Pediatric Oncology Branch, National Cancer Institute, National Institutes of Health, Bethesda, MD, USA

## Abstract

**Background:**

Whole genome amplification (WGA) promises to eliminate practical molecular genetic analysis limitations associated with genomic DNA (gDNA) quantity. We evaluated the performance of multiple displacement amplification (MDA) WGA using gDNA extracted from lymphoblastoid cell lines (N = 27) with a range of starting gDNA input of 1–200 ng into the WGA reaction. Yield and composition analysis of whole genome amplified DNA (wgaDNA) was performed using three DNA quantification methods (OD, PicoGreen^® ^and RT-PCR). Two panels of N = 15 STR (using the AmpF*l*STR^® ^Identifiler^® ^panel) and N = 49 SNP (TaqMan^®^) genotyping assays were performed on each gDNA and wgaDNA sample in duplicate. gDNA and wgaDNA masses of 1, 4 and 20 ng were used in the SNP assays to evaluate the effects of DNA mass on SNP genotyping assay performance. A total of N = 6,880 STR and N = 56,448 SNP genotype attempts provided adequate power to detect differences in STR and SNP genotyping performance between gDNA and wgaDNA, and among wgaDNA produced from a range of gDNA templates inputs.

**Results:**

The proportion of double-stranded wgaDNA and human-specific PCR amplifiable wgaDNA increased with increased gDNA input into the WGA reaction. Increased amounts of gDNA input into the WGA reaction improved wgaDNA genotyping performance. Genotype completion or genotype concordance rates of wgaDNA produced from all gDNA input levels were observed to be reduced compared to gDNA, although the reduction was not always statistically significant. Reduced wgaDNA genotyping performance was primarily due to the increased variance of allelic amplification, resulting in loss of heterozygosity or increased undetermined genotypes. MDA WGA produces wgaDNA from no template control samples; such samples exhibited substantial false-positive genotyping rates.

**Conclusion:**

The amount of gDNA input into the MDA WGA reaction is a critical determinant of genotyping performance of wgaDNA. At least 10 ng of lymphoblastoid gDNA input into MDA WGA is required to obtain wgaDNA TaqMan^® ^SNP assay genotyping performance equivalent to that of gDNA. Over 100 ng of lymphoblastoid gDNA input into MDA WGA is required to obtain optimal STR genotyping performance using the AmpF*l*STR^® ^Identifiler^® ^panel from wgaDNA equivalent to that of gDNA.

## Background

The potential for the molecular analysis of human genetic material has increased enormously with the availability of the human genome sequence, SNP identification efforts and the development of high-throughput genotyping platforms [1]. The expanding demand for single nucleotide polymorphism (SNP) genotyping is a consequence of the recognition that many SNPs will need to be analyzed to characterize the effects of genes on complex disorders [2], especially when performing whole genome association studies [3]. With notable exceptions [4], total DNA requirements for genotyping will increase as the number of loci investigated expands, despite increased efficiency of individual genotyping assays. Whole genome amplification (WGA) is an *in vitro *procedure to amplify a genomic DNA (gDNA) sample to generate amplified DNA (wgaDNA) for further molecular genetic analyses, and has been considered by some as a potential solution to the problem of limiting gDNA availability. While PCR-based methods of WGA have been under continuous development for over a decade [5,6], recent application of a highly processive φ29 DNA polymerase [7], has enabled multiple displacement amplification (MDA) WGA, an isothermal, hyperbranching amplification method, with a low level of locus or allelic bias [8]. Dean [8] and Lovmar [9] have evaluated the genotyping performance of MDA WGA using a range of genomic DNA inputs (0.3, 3, 30 and 300 ng, and 0.003, 0.03, 0.3 and 3 ng, respectively). Both authors focused attention in their evaluation of genotyping performance on genotyping wgaDNA derived from 3 ng of genomic DNA template. Lasken and Egholm [10] have recommended 10–100 ng of undegraded gDNA template in the MDA WGA reaction to avoid stochastic amplification. The present study has characterized the yield, composition and genotyping performance of wgaDNA produced from lymphoblast gDNA templates of 1, 10, 25, 50, 100 and 200 ng. Three DNA quantification methods, two genotyping methods, and adequate numbers of genotyping assays and DNA samples were used to detect significant differences in the yield, composition and genotyping performance of the wgaDNA produced from this range of gDNA inputs and to provide additional recommendations on the amounts of gDNA template to be used in the MDA WGA reaction.

## Results

### WGA reaction yield

**Figure 1:**
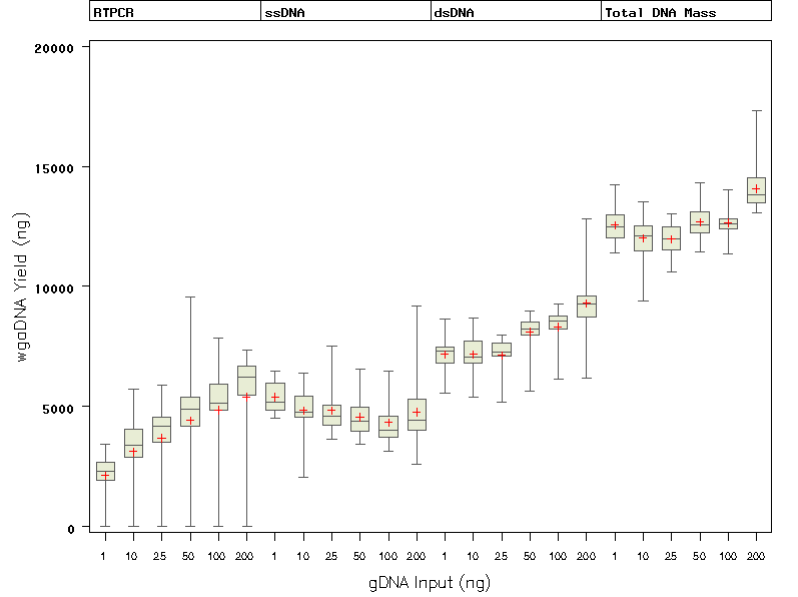
**Yield of DNA components of wgaDNA by gDNA input into WGA. **Mean ("+"), Median (middle bar), lower and upper quartile (lower and upper end of box), and minimum and maximum of BRCA1 locus equivalents, ssDNA, dsDNA and total DNA.

The yield of *H. sapiens *PCR-amplifiable (hereafter "RT-PCR") DNA, ssDNA, dsDNA and total DNA in wgaDNA by gDNA input mass is presented in Figure 1. RT-PCR DNA yield increased significantly as gDNA input increased at each level (all p values ≤ 0.02), where the proportion of the total wgaDNA represented by the RT-PCR DNA increased from 20% to 46%, at 1 to 200 ng gDNA input into the WGA reaction, respectively. The yield of ssDNA decreased, and that of dsDNA increased, as the gDNA input into the WGA reaction was increased. The variability in wgaDNA yield by wgaDNA component was least for total DNA and dsDNA yield, greatest for RT-PCR yield, and intermediate for ssDNA yield.

### Genetic profiling with AmpFlSTR^® ^Identifiler^® ^assay (N = 15 STR and AMEL)

gDNA exhibited STR genotype completion and concordance rates of 100%, which were significantly greater than the completion rate exhibited by wgaDNA produced from 1 ng gDNA input and the wgaDNA concordance produced from all gDNA inputs, respectively (Table 1). wgaDNA produced from 1 ng gDNA input exhibited significantly lower STR genotype completion and concordance rates than did wgaDNA produced from other gDNA inputs, while wgaDNA produced from 200 ng gDNA input exhibited STR genotyping completion and concordance rates similar, but not identical to gDNA. 98% of wgaDNA STR genotypes discordant with gDNA genotypes were homozygote genotypes, reflecting loss of heterozygosity. There was a trend for preferential loss of shorter alleles (129 short alleles/232 total alleles, p = 0.088), but only for wgaDNA produced from 1 ng of gDNA was this significant (90 short alleles/145 total alleles, p = 0.0037). Peak heights were significantly and negatively correlated with discordance for all gDNA inputs, and for 1, 10 and 100 ng gDNA inputs separately (Spearman r = -0.58, -0.64, -0.55 and -0.62, with p values <0.0001, = 0.008, = 0.025 and = 0.001, respectively, data not shown), and peak height ratios of concordant heterozygote wgaDNA genotypes (from wgaDNA produced from 1 and 50 ng gDNA inputs) were significantly higher than those from gDNA genotypes (Wilcoxon's p values ≤ 0.03, data not shown).

The rate of no amplification and discordant genotypes per STR locus was 0.8% and 4%, respectively. Five STR loci (*TPOX, FGA*, D7S820, D13S317 and D18S51) accounted for the majority of STR no amplification failures (82%) and discordant (56%) genotypes following WGA (Table 2). The discordance rate for *AMEL *genotypes for all wgaDNA strata was 0.15%, but was 0.73% for wgaDNA produced from 1 ng gDNA input (Table 2). Composite genotype quality (GQ) scores for gDNA heterozygote and homozygote concordant genotypes were significantly better (fewer genotypes in the poorer quality categories) than for concordant wgaDNA heterozygote genotypes at all gDNA input levels and for concordant wgaDNA homozygote genotypes produced from 1, 100 and 200 ng gDNA input levels, respectively (Table 3). wgaDNA heterozygote and homozygote concordant genotypes produced from 1 ng gDNA input exhibited significantly reduced GQ scores compared to wgaDNA heterozygote and homozygote concordant genotypes produced from all other gDNA input levels, except for wgaDNA homozygote concordant genotypes produced from 200 ng gDNA (Table 3). GQ scores of discordant homozygote wgaDNA genotypes were significantly worse than those for concordant homozygote wgaDNA genotypes at all gDNA input levels except 50 ng (p = 0.02 for 25 ng gDNA input, all other p < 0.0001, data not shown).

### SNP genotyping with the TaqMan^® ^assay (N = 49 SNPs)

Results of genotyping using N = 49 TaqMan^® ^SNP genotyping assays with 1, 4 and 20 ng of gDNA and wgaDNA using 1, 10, 25, 50, 100 and 200 ng of gDNA input into the WGA reaction are summarized in Table 4. We observed a TaqMan^® ^SNP genotype completion rate of >99.55%, an undetermined rate of <0.45%, zero discordant genotypes and zero "no amplification failures" in 7938 attempted TaqMan^® ^SNP genotypes using gDNA template in the TaqMan^® ^SNP assay. No significant differences in genotyping performance between gDNA template inputs into the TaqMan^® ^SNP assay were observed (Table 4). In pairwise tests, gDNA exhibited a significantly higher TaqMan^® ^SNP genotype completion rate due to significantly decreased undetermined TaqMan^® ^SNP genotypes, compared to wgaDNA produced from 1 ng of gDNA input for all wgaDNA template inputs into TaqMan^® ^SNP genotyping, and when compared to wgaDNA produced from 50 and 100 ng of gDNA input when using 1 or 4 ng of wgaDNA template input into the TaqMan^® ^SNP assay. Over all gDNA and wgaDNA strata, we observed significantly reduced SNP genotyping performance when using 1 ng of gDNA or wgaDNA in TaqMan^® ^SNP genotyping assays with respect to completion rate, due to a significant increase in the undetermined genotype rate (Table 4). However, genotype concordance rates were not significantly different among the three DNA (gDNA or wgaDNA) input levels into the TaqMan^® ^SNP assay, although there was a significant decrease in the concordance rate of 1 ng wgaDNA produced from 1 ng gDNA into the TaqMan^® ^SNP assay, when compared to 1 ng wgaDNA produced from 10, 50 and 100 ng of gDNA input (Table 4).

### Predictors of wgaDNA SNP genotyping performance

We were interested to identify parameters from the Core Genotyping Facility's standard DNA sample handling protocol that might be predictive of the SNP genotyping performance of wgaDNA. We performed exploratory correlation analysis among measures of wgaDNA yield (RT-PCR, ssDNA, dsDNA, total DNA, ratio of RT-PCR to dsDNA) and genotyping performance (concordance and completion rates for AmpF*l*STR^® ^Identifiler™ and TaqMan^® ^SNP assay genotyping) within gDNA input strata. Measures of wgaDNA yield (especially the ratio of RT-PCR to dsDNA and total DNA) and genotyping performance were observed to be highly correlated with one another (92%, 3% and 5% of 162 pairwise correlations were statistically significant, trending and non-significant, respectively). We then performed linear regression analysis with the dependent variables "SNP completion rate" and "SNP concordance rate", in order to identify WGA reaction, wgaDNA yield and STR genotyping performance factors that are significantly associated with wgaDNA SNP genotyping performance. Independent variables included: gDNA input, STR completion rate, concordance rate, GQ score, peak height, and RT-PCR wgaDNA yield. "STR completion rate" was a highly significant factor in both SNP rate models (p < 0.0001), and "STR concordance rate" and "GQ score" were significant factors in the SNP concordance rate model (p = 0.0008 and 0.045, respectively). The variable "gDNA input" into the WGA reaction was significant only in those models incorporating the 1 ng gDNA input strata.

### WGA yield and genotyping performance with no template control (NTC) samples

No template control (NTC) input samples, i.e., where no gDNA was used in the WGA reaction, yielded substantial amounts of wgaDNA, similar in quantity to the total wgaDNA obtained with gDNA inputs, but with a substantially higher proportion of ssDNA than with gDNA inputs (Table 5). The wgaDNA produced from the NTC samples in the 1, 50 and 100 ng gDNA input strata exhibited mean RT-PCR results that were greater than zero (Table 5). We observed N = 35 STR peaks with a signal strength > 50 RFUs that fell within the expected base-pair range of an AmpF*l*STR^® ^Identifiler™ locus allele from the wgaDNA produced from the NTC gDNA and wgaDNA samples for an overall false positive STR genotyping rate of 4.2% (Table 5). While these false positive STR peaks fulfill the criteria for valid AmpF*l*STR^® ^Identifiler™ STR alleles, they are characterized by low heterozygosity (2 observed versus 26 expected heterozygote genotypes), moderate signal strength (median amplitude = 357 RFUs), and representation of 12 out of 15 AmpF*l*STR^® ^Identifiler™ STR loci. The 50 and 100 ng gDNA input strata (Table 5) and three STR loci (D2S1338, D8S1179 and FGA)account for the majority (66% and 51%, respectively) of the wgaDNA false positive STR genotypes produced from the NTC samples.

In N = 7056 TaqMan SNP genotype attempts with wgaDNA produced from the NTC samples, 80%, 14.5% and 5.5% of the resulting datapoints were incorporated into the no amplification (NTC) cluster, into a genotype cluster ("false positive SNP genotypes"), and into the undetermined genotype space of the two color TaqMan^® ^SNP genotyping assay plot, respectively (Table 6). The number of false positive and undetermined SNP genotypes from the wgaDNA produced from the NTC samples increased significantly with increasing amounts of wgaDNA input into the TaqMan^® ^SNP assay (Table 6). The majority (96.4%) of these false positive SNP genotypes from NTC samples were homozygotes (Table 6), significantly more allele 2 alleles were observed than allele 1 alleles (Table 6), and all N = 49 TaqMan^® ^SNP assays exhibited false positive SNP genotypes (data not shown). wgaDNA NTC samples from the gDNA input strata of 1, 50 and 100 ng exhibited significantly greater numbers of false positive and undetermined SNP genotypes than did the wgaDNA NTC samples from the gDNA input strata of 10, 25 and 200 ng (Table 6).

## Discussion

### wgaDNA may not be suitable for STR genotyping

wgaDNA STR genotyping completion rates reach that of gDNA at the 10 ng gDNA input level into WGA. However, the wgaDNA STR concordance rate is significantly worse than that of gDNA, even with 200 ng of gDNA input into the WGA reaction (Table 1). Thus, the use of MDA wgaDNA for accurate STR genotyping will require larger amounts of input gDNA into the WGA than have been recommended in the past [8,10]. In the absence of sufficient gDNA template for MDA WGA, investigators face the tradeoff of no data, or data with increased loss of heterozygosity, such as that observed with MDA wgaDNA produced from low mass gDNA templates [11,12]. Development of laboratory and data analysis protocols optimized for STR genotyping of MDA wgaDNA may be required before MDA wgaDNA can be routinely used for STR genotyping. Thus, it might be prudent to adjust genotype analysis algorithms before application of the AmpF*l*STR^® ^Identifiler™ panel to wgaDNA for forensic purposes, as has been recommended for the analysis of STR profiles from highly limited unamplified gDNA template [13], or to utilize analysis methods that incorporate STR genotyping error, as has been recommended for the analysis of STR linkage scan data [14].

### wgaDNA is a suitable template for SNP genotyping

wgaDNA produced from ≥ 10 ng of gDNA input into the WGA reaction exhibits robust wgaDNA TaqMan^® ^SNP assay genotyping performance rates, similar to that of gDNA TaqMan^® ^SNP assay genotyping performance rates. 1 ng of wgaDNA template into the TaqMan^® ^SNP assay exhibits significantly reduced TaqMan^® ^SNP assay genotyping performance compared to both 4 and 20 ng wgaDNA templates into the TaqMan^® ^SNP assay. 4 ng wgaDNA template into the TaqMan^® ^SNP assay exhibits a significantly increased no amplification rate over both 1 and 20 ng wgaDNA templates, although no amplification rates are very low (all <0.01%) for all three wgaDNA template inputs into the TaqMan^® ^SNP assay. These results suggest that optimal TaqMan^® ^SNP assay genotyping performance, i.e., minimal wgaDNA TaqMan^® ^no amplification and undetermined genotyping rates, should be expected for wgaDNA inputs greater than 4 ng.

### False positive NTC sample SNP genotypes

A non-zero RT-PCR yield and significantly increased numbers of observed false positive genotypes in wgaDNA from NTC samples in the 1, 50 and 100 ng gDNA input strata are consistent with human gDNA contamination of these gDNA input strata. However, we also observed significantly more false positive and undetermined SNP assay genotypes in each of the 10, 25 and 200 ng gDNA input strata (the apparently uncontaminated strata) than in the gDNA strata (all p < 0.0001), concordant with the hypothesis that a portion of the NTC TaqMan^® ^genotypes may be due to degradation of TaqMan^® ^SNP assay reagents. Thus, contamination of NTC samples with gDNA and TaqMan^® ^SNP assay probe oligonucleotide degradation during the genotyping of wgaDNA are both associated with false-positive TaqMan^® ^SNP assay genotypes.

### Limitations

This study is distinguished by the use of multiple assays to estimate wgaDNA yield and composition, the use of STR and SNP genotyping assays that have been validated by sequencing the same DNA samples used in this study, and the use of an adequate number of samples and assays to provide statistical power to detect small differences in the genotyping performance of wgaDNA and gDNA, when using 1–200 ng of gDNA as template in the WGA reaction. Nevertheless, there are limitations, with respect to generalizing to all gDNA templates, MDA protocols and genotyping methods, respectively.

The gDNA used in this study was extracted from lymphoblasts and samples from most studies are unlikely to be of such high quality. Using a model system to evaluate the effect of significant gDNA degradation on the WGA reaction, it has been shown that MDA wgaDNA produced from irradiated gDNA exhibits significantly reduced yield and genotyping performance compared to MDA wgaDNA produced from unirradiated gDNA [15]. The yield and genotyping differences observed in wgaDNA produced from high quality (this study) and low quality [15] gDNA samples suggest that those gDNA samples with DNA extraction, storage and usage histories that have reduced concentrations of high molecular weight DNA in the sample are likely to exhibit less than optimal MDA wgaDNA yield and genotyping performance.

While only one commercially available MDA WGA protocol was used in this study, we have evaluated two MDA WGA protocols on gDNA extracted from multiple tissue types, and no systematic significant differences in genotyping performance between the two MDA WGA protocols was observed [16]. STR and SNP genotyping performance of MDA wgaDNA derived from 4 ng of gDNA input in that study is seen to be intermediate between the genotyping performance of MDA wgaDNA produced from 1 ng and 10 ng in this study. Alternative WGA technologies that can prepare wgaDNA of acceptable quality from gDNA with reduced complexity or concentration may be required for some degraded gDNA samples. For example, PCR-based methods that reduce genome complexity before amplification are one approach [6,17], and methods that combines genome circularization with φ29 DNA polymerase are another [18].

Finally, we applied two commonly used genotyping methods to evaluate the genotyping performance of wgaDNA in this study. Different genotyping technologies may be better suited to produce optimal genotyping performance with wgaDNA than the two we evaluated. For STRs, genotyping panels designed for linkage scanning usually employ lower levels of multiplexing and use larger amounts of DNA template than do STR panels designed for forensic analysis, such as the AmpF*l*STR^® ^Identifiler™. E.g., reported MDA wgaDNA STR genotype discordance rates using linkage scan STR panels [19,20] and forensic STR panels [15,16,21] range from ~0% to ~6% and the average rate of the five studies cited (2.0%) is similar to the rates observed in this study. For SNPs, those genotyping technologies with redundant data sampling for SNP genotype determination, such as minisequencing [22], the Golden Gate™ assay [23] or the GeneChip^® ^variant detection array [24], may be more resistant to SNP genotype failure when genotyping wgaDNA [9,25,26] than those SNP genotyping technologies with single data point genotype determination [27]. However, in a recent direct comparison of Golden Gate™, TaqMan^® ^and Invader™ SNP assays, with gDNA extracted from lymphoblasts using an organic extraction method and MDA wgaDNA produced from 20 ng of this gDNA, the Golden Gate™ assay exhibited a higher exclusion rate of DNA samples, and a higher completion rate and lower concordance rate on the remaining samples, than exhibited by the TaqMan^® ^and Invader™ SNP assays [28]. For all three SNP genotyping technologies evaluated, the genotyping performance of gDNA was observed to be significantly better than that of MDA wgaDNA [28].

## Conclusion

We have evaluated the yield, composition and genotyping performance of wgaDNA based on a range of high-quality lymphoblastoid gDNA templates between 1 and 200 ng in order to provide empirical data on the performance of MDA WGA technology. A detailed analysis of the observed genotyping failures has been performed to facilitate an understanding of the reduction in genotyping performance likely to be observed when genotyping wgaDNA produced from a range of gDNA inputs. Increasing gDNA input from 1 – 200 ng in the MDA WGA reaction improves the yield of *H. sapiens *PCR-amplifiable DNA and improves the genotyping performance of the AmpF*l*STR^® ^Identifiler^® ^assay. More than 100 ng of high quality gDNA template into the MDA WGA reaction is required in order to observe MDA wgaDNA AmpF*l*STR^® ^Identifiler^® ^STR genotyping performance similar to that observed with gDNA. At least 10 ng of high quality lymphoblastoid gDNA template into the WGA reaction is required to observe optimal TaqMan^® ^SNP genotyping performance from MDA wgaDNA.

## Methods

### gDNA samples

N = 22 lymphoblast genomic DNA (gDNA) samples were obtained directly from the Coriell Cell Repository (Camden, NJ); these samples were from individuals within the SNP500 Cancer dataset [29]. Each gDNA was quantified by UV spectroscopy, the PicoGreen^® ^assay (Molecular Probes, Eugene, OR), and a Real-Time (RT) TaqMan^® ^assay specific to human DNA [30]. Five of twenty-two Coriell Cell Repository lymphoblast gDNA samples were replicated for a total of N = 27 lymphoblast gDNA samples subjected to WGA and post-WGA analysis in order to increase statistical power to detect genotyping error.

### Whole genome amplification

1, 10, 25, 50, 100 and 200 ng of each gDNA sample was used as template and amplified according to the GenomiPhi™ WGA protocol (1X). The 200 ng gDNA template sample was amplified separately after evaluation of the genotyping performance of the wgaDNA produced from 1 – 100 ng gDNA. Each gDNA sample was subjected to the WGA protocol once; four no gDNA template controls (NTC) reactions were included at each gDNA input level. wgaDNA was quantified with OD_260_, PicoGreen^® ^and RT-PCR, as was performed for gDNA. The concentrations of ssDNA, dsDNA, total DNA and human-specific PCR amplifiable (RT-PCR) DNA in the wgaDNA samples were estimated as described [16].

### AmpFlSTR^® ^Identifiler^® ^assay

300 pg of dsDNA (both gDNA and wgaDNA, as determined by PicoGreen^®^) was used as template DNA for AmpF*l*STR^® ^Identifiler^® ^assay (Applied Biosystems Inc., Foster City, CA), and scoring of alleles, assignment of Genotype Quality scores and calculation of genotype failure rates were performed as described [16]. Peak height ratio distributions at a signal strength threshold of = 50 RFUs were evaluated for normality and differences between assigned and observed GQ score category distributions evaluated using Wilcoxon's rank sum test and contingency table analyses.

### TaqMan^® ^SNP genotype assays

N = 49 TaqMan^® ^(Applied Biosystems Inc., Foster City, CA) genotyping assays from the publicly available SNP500 Cancer Database portfolio [29] were chosen as described [16]. 1.0, 4.0 and 20.0 ng of dsDNA (both gDNA and wgaDNA, as determined by PicoGreen^®^) was used as template for genotyping using the N = 49 TaqMan^® ^assays. Reaction and cycling conditions, control samples, fluorescence detection and genotype cluster assignment were performed as described [16]. SNP genotype completion, undetermined genotype, no amplification, and discordance rates were calculated, with the wgaDNA discordance rate calculated to be the number of instances in which a wgaDNA SNP genotype differed from the scored gDNA SNP genotype. Differences in rates were evaluated using contingency table analyses.

### Data management and analysis

Data was managed using a Sapphire Laboratory Information Management System (LabVantage, New Brunswick, NJ), exported in Microsoft Excel (Redmond, WA) and statistical analyses (descriptive statistics and tests of normality, distribution and correlation) were performed using SAS (Cary, NC) software. Tests of proportion, correlation, etc., are considered significant at a Type I error level of 0.05, with additional information on p values provided if appropriate.

## Authors' contributions

AWB conceived of the study and drafted the manuscript. AWB, YQ, KAH and RAW participated in the experimental design. KAH designed and performed the amplification, quantification and genotyping experiments. YQ performed the statistical analysis. AWB, YQ, KAH and RAW participated in the interpretation of the data, and YQ, KAH, RW and SJC helped to draft the manuscript. All authors read and approved the final manuscript.

## Figures and Tables

**Table 1:** STR genotyping performance

**gDNA Input (ng)**	**Completed^1^**	**% Completion**	**No Amplification**	**GQ<0.25^2^**	**Concordant**	**% Concordance**	**Discordant Genotypes**	**% Discordance**
gDNA	864	100.0^3^	0	13	851	100.0^4^	0	0.0
1	821	95.0	43	70	638	80.1	150	19.0
10	862	99.8	2	9	830	97.1	25	2.9
25	863	99.9	1	22	823	97.7	19	2.3
50	863	99.9	1	15	836	98.6	12	1.4
100	861	99.7	3	11	826	96.8	27	3.2
200	864	100.0	0	0	858	99.3	6	0.7

^1^N = 27 gDNA or wgaDNA samples were genotyped in duplicate using the AmpFlSTR^® ^Identifiler^® ^assay for N = 864 attempted genotypes/sample. ^2^A Genotype Quality Score (GQ) of <0.25 indicates a STR genotype with a Genotype Quality Score beloew the calling threshold of GeneMapper v3.0 software. ^3^gDNA exhibited significantly greater STR genotype completion rate than did 1 ng gDNA input (p < 0.001). ^4^gDNA exhibited significantly greater STR genotype concordance rates compared to wgaDNA (p < 0.0001, except for 50 ng gDNA input, with p = 0.001, and 200 ng gDNA input, p = 0.03).

**Table 2:** STR genotyping failures by locus

**gDNA Input (ng)**	**Failure Type**	**Genotype Failures^1^**	**L1^2^**	**L2**	**L3**	**L4**	**L5**	**L6**	**L7**	**L8**	**L9**	**L10**	**L11**	**L12**	**L13**	**L14**	**L15**	**L16**
gDNA	No Amp.	0	0	0	0	0	0	0	0	0	0	0	0	0	0	0	0	0
	Disc.	0	0	0	0	0	0	0	0	0	0	0	0	0	0	0	0	0
1	No Amp.	43	5	0	0	3	0	0	7	0	0	3	5	0	15	1	1	3
	Disc.	150	11	3	9	10	4	5	13	9	2	5	22	9	24	7	11	6
10	No Amp.	2	1	0	0	1	0	0	0	0	0	0	0	0	0	0	0	0
	Disc.	25	2	0	0	5	1	0	2	0	1	0	7	1	5	0	0	1
25	No Amp.	1	0	0	0	0	0	0	0	0	0	0	0	0	1	0	0	0
	Disc.	19	4	1	1	0	0	2	2	0	0	0	2	0	3	2	1	1
50	No Amp.	1	0	0	0	0	0	0	0	0	0	0	0	0	1	0	0	0
	Disc.	12	0	0	1	3	0	0	1	2	0	0	0	0	2	1	1	1
100	No Amp.	3	0	0	0	1	0	0	0	0	0	0	0	1	1	0	0	0
	Disc.	27	5	5	0	2	0	1	2	1	6	0	0	2	1	2	0	0
200	No Amp.	0	0	0	0	0	0	0	0	0	0	0	0	0	0	0	0	0
	Disc.	6	1	1	0	2	0	0	0	0	0	0	2	0	0	0	0	0
			
	**No Amp.**	**Total**	23	10	11	22	5	8	20	12	9	5	33	12	35	12	13	9
	**Discordant**	**Total**	6	0	0	5	0	0	7	0	0	3	5	1	18	1	1	3

^1^The number of attempted genotypes for all DNA inputs is N = 864. No amplification (No Amp.) genotypes reduce the number of genotypes available for concordance analysis. ^2^Loci 1–16 = *TPOX*, D2S1338, D3S1358, *FGA*, D5S818, *CSF1PO*, D7S820, D8S1179, *TH01*, *vWA*, D13S2317, D16S539, D18S51, D19S433, D21S11, *AMEL*.

**Table 3:** Genotype Quality (GQ) scores of concordant STR genotypes

**gDNA Input (ng)**	**GQ^1^Category, Heterozygotes**			
	**I**	**II**	**III**	**Total**
gDNA	5	53	580	638^2^
1	43	137	256	436^3^
10	27	140	451	618
25	26	126	463	615
50	21	130	474	625
100	25	110	482	617
200	78	31	537	646
	
Total	225	727	3243	4195

**gDNA Input (ng)**	**GQ Category, Homozygotes**			
	**I**	**II**	**III**	**Total**

gDNA	0	9	204	213^4^
1	9	25	168	202^5^
10	2	8	202	212
25	1	8	199	208
50	4	11	196	211
100	8	10	191	209
200	8	20	184	212
	
Total	32	91	1344	1467

^1^Category I = GQ scores, originally ≥ 0.25 and <0.40, that have been successfully edited; Category II = GQ scores ≥ 0.40 and ≤ 0.50; Category III = GQ scores ≥ 0.79 and ≤ 0.90. ^2^GQ score distribution, heterozygote genotypes, gDNA versus all wgaDNA, p < 0.0001. ^3^GQ score distribution, heterozygote genotypes, wgaDNA produced from 1 ng gDNA input versus other gDNA inputs, p < 0.0001.^4^GQ score distribution, homozygote genotypes, gDNA versus wgaDNA produced from 1, 100, 200 ng gDNA input, p < 0.0001, 0.01, 0.0001, respectively. ^5^GQ score distribution, homozygote genotypes, wgaDNA produced from 1 ng gDNA input versus 10, 25, 50, 100 ng gDNA inputs, p = 0.0003, 0.0001, 0.009, 0.02, respectively.

**Table 4:** SNP genotyping performance

**gDNA Input (ng)**	**Completed Genotypes**^1^	**% Completion**	**Undeter. Genotypes**	**% Undeter.**	**No Amp.**	**% No Amp.**	**Concordant Genotypes**	**% Concordance**	**Discordant Genotypes**	**% Discordance**
**1 ng gDNA or wgaDNA input into TaqMan^® ^SNP genotype assay**^2,3^										

gDNA	2636	99.62	10	0.38	0	0	2636	100.00	0	0.00
1	2552^4^	96.45	94^5^	3.55	0	0	2546	99.76	6^8^	0.24
10	2627	99.28	19	0.72	0	0	2627	100.00	0	0.00
25	2636	99.62	10	0.38	0	0	2635	99.96	1	0.04
50	2614	98.79	31^6^	1.17	1	0.04	2614	100.00	0	0.00
100	2620	99.02	25^6^	0.94	1	0.04	2620	100.00	0	0.00
200	2623	98.07	21	0.79	2	0.08	2622	99.96	1	0.04

**4 ng gDNA or wgaDNA into TaqMan^® ^SNP genotype assay**^4^										

gDNA	2634	99.55	12	0.45	0	0.00	2634	100.00	0	0.00
1	2589^4^	97.85	55^5^	2.08	2	0.08	2586	99.88	3	0.12
10	2628	99.32	17	0.64	1	0.04	2627	99.96	1	0.04
25	2629	99.36	14	0.53	3	0.11	2629	100.00	0	0.00
50	2621	99.06	23^5^	0.87	2	0.08	2619	99.92	2	0.08
100	2619	98.98	20^5^	0.76	7^7^	0.26	2617	99.92	2	0.08
200	2635	99.4	10	0.38	1	0.04	2632	99.89	3	0.11

**20 ng gDNA or wgaDNA into TaqMan^® ^SNP genotype assay**										

gDNA	2637	99.66	9	0.34	0	0	2637	100.00	0	0.00
1	2569^4^	97.09	77^5^	2.91	0	0	2566	99.88	3	0.12
10	2633	99.51	13	0.49	0	0	2633	100.00	0	0.00
25	2639	99.74	7	0.26	0	0	2638	99.96	1	0.04
50	2633	99.51	13	0.49	0	0	2632	99.96	1	0.04
100	2630	99.40	16	0.60	0	0	2629	99.96	1	0.04
200	2635	99.55	11	0.42	0	0	2632	99.89	3	0.11

^1^There were N = 27 samples gentoyped in duplicate at N = 49 SNPs for N = 2,646 attempted genotypes/sample. ^2^1 versus 4 ng gDNA or wgaDNA input, SNP genotyping rates: completion, p = 0.0178; undetermined, p = 0.0022; no amplification, p = 0.0139; concordance, p = n.s. ^3^1 versus 20 ng gDNA or wgaDNA input, SNP genotyping rates: completion, p = 0.0004; undetermined, p = 0.0008; no amplification, p = n.s.; concordance, p = n.s. ^4^4 versus 20 ng gDNA or wgaDNA input, SNP genotyping rates: completion, p = n.s.; undetermined, p = n.s.; no amplification, p = 0.0002; concordance, p = n.s. ^5^gDNA versus wgaDNA produced from 1 ng gDNA input, SNP genotype completion rates, p < 0.0001. ^6^gDNA versus wgaDNA produced from 50 and 100 ng gDNA input, p ≤ 0.05. ^7^gDNA versus wgaDNA produced from 100 ng gDNA input, p = 0.02. ^8^wgaDNA produced from 1 ng versus gDNA and wgaDNA produced from 10, 50 and 100 ng of gDNA input, p ≤ 0.014.

**Table 5:** Yield and STR genotypes from NTC samples

**gDNA Input (ng)**	**Yield**			
	**N**	**Mean RT-PCR (ng)**	**Median ssDNA (%)**	**Median total DNA (ng)**
gDNA	-	-	-	-
1	4	135	51.5	12230
10	4	0	50.8	11771
25	4	0	55	12340
50	4	24	45	12395
100	4	220	48.6	12686
200	4	0	54	14879

	**STR**			
	
**gDNA Input (ng)**	**N**	**False Positive Rate (%)**^1^	**Mean Height Allele 1**	**Mean Height Allele 2**

gDNA	64	6	93	93
1	128	5	2185	2199
10	128	0	-	-
25	128	0	-	-
50	128	9^2^	375	393
100	128	9^2^	571	571
200	128	2	2360	2360

^1^The number of "attempted" AmpFlSTR^® ^Identifiler™ panel genotypes using gDNA NTCs and wgaDNA from NTC samples is 832 [two NTC DNA samples genotyped for gDNA in duplicate (64 possible genotype bins), and four wgaDNA samples from NTCs for each gDNA input level genotyped in duplicate (768 possible genotype bins)]. ^2^There were significantly more false positive STR genotypes in the wgaDNA produced from 50 ng and 100 ng gDNA inputs compared to the wgaDNA produced from 200 ng gDNA input (p = 0.01 and 0.02).

**Table 6:** SNP genotypes from NTC samples

**gDNA Input (ng)**	**Allele 1**	**Allele 2**	**Both**	**No Amp.**	**Undeter.**	**Total**
**1 ng gDNA or wgaDNA input into TaqMan^® ^SNP genotype assay**^1^						

gDNA	0	2	0	385	5	392
1^3^	27	29	2	315	19	392
10	6	12	1	362	11	392
25	6	10	0	364	12	392
50^3^	38	49	5	266	34	392
100^3^	26	48	2	289	27	392
200	18	20	0	351	3	392
	
Total	121	170^2^	10	2332	111	2744

**4 ng gDNA or wgaDNA input into TaqMan^® ^SNP genotype assay**^1^						

gDNA	1	2	0	381	8	392
1^3^	39	36	2	292	23	392
10	7	7	0	360	18	392
25	6	18	1	349	18	392
50^3^	50	43	0	275	24	392
100^3^	26	43	2	274	47	392
200	26	38	3	316	9	392
	
Total	155	187^2^	8	2247	147	2744

**20 ng gDNA or wgaDNA input into TaqMan^® ^SNP genotype assay**^1^						

gDNA	0	1	0	383	8	392
1^3^	29	37	2	297	27	392
10	11	15	1	346	19	392
25	8	9	0	364	11	392
50^3^	37	54	4	263	34	392
100^3^	25	55	2	277	33	392
200	38	42	10	284	18	392
	
Total	148	213^2^	19	2214	150	2744

^1^A significant increase in the number of false positive and undetermined SNP genotypes is observed with increasing amounts of wgaDNA template: 11.0%, 12.8% and 13.8% for 1, 4 and 20 ng wgaDNA input, respectively, p = 0.045, 1 versus 4 ng wgaDNA input, and p = 0.0014, 1 versus 20 ng wgaDNA input, p = 0.0046 test for trend, 1 vs. 4 vs 20 ng wgaDNA input, for false positive genotypes; 4.0%, 5.34% and 5.47%, respectively, p = 0.026, 1 versus 4 ng, and p = 0.0159, 1 versus 20 ng, p = 0.0153 for trend, for undetermined genotypes. ^2^Significantly more allele 2 NTC TaqMan^® ^SNP assay alleles were observed than allele 1 NTC TaqMan^® ^assay alleles (p = 0.006, 0.091 and 0.001 for 1, 4 and 20 ng gDNA and wgaDNA input into the TaqMan^® ^SNP assays, respectively), where the fluorescent label was 6-Fam for allele 1, and Vic for allele 2, in all the TaqMan^® ^SNP assays in this study. ^3^The gDNA input strata of 1, 50 and 100 ng exhibited significantly greater numbers of false positive and undetermined SNP genotypes than did the gDNA input strata of 10, 25 and 200 ng (p < 0.0001).
